# Supplementary material for: The social function of the feeling and expression of guilt
Source: R Soc Open Sci. 2020 Dec 9;7(12):200617. doi: 10.1098/rsos.200617 (PMC7813227; doi:10.1098/rsos.200617)
Supplement: Supplementary Material - Procedure [file rsos200617supp1.docx]

# Supplementary Material

## Participants

**Demographics.** Two hundred and eighteen participants were recruited as pairs; participants were given the opportunity to either sign up with a friend or sign up alone and be paired up with a stranger (only 36 participants signed up alone to be paired up). The quality of friendship (measured during the game; see below for details) was normally distributed in our sample; we thus considered friendship as a continuous independent variable. Participants were recruited based on an opportunistic sampling method and were either part of the University community (students and staff members) or part of the general public. Three participants were excluded from the study as they did not complete all the steps required and we were therefore unable to use their final data for analysis (final sample = 215). In the end, one hundred and seven participants took part as Player 1 and one hundred and eight participants took part as Player 2 (see procedure for details).

Participants had various ethnicities and nationalities, constituting a sample made of individuals with various places of origin [[PoO - see SMTable 1 for details; 1](#_ENREF_1)]. Participants were clustered into two regions for PoO: Europe and East Asia. One hundred and twenty-eight participants had European nationalities (81 female; mean age = 25.30, *SD* = 8.35) and 87 participants had East-Asian nationalites (including some participants with dual ethnicities; 55 female; mean age = 23.44, *SD* = 5.46; see SMTable 1 for details). Ethnic identity was established by self-identification: we asked participants to indicate the ethnical group they identified as. PoO was added to our analysis to control for the potential influence of culture but was not considered as a variable.

All participants were compensated £10 for their time. The experiment lasted for approximately 50 minutes. The projects have been reviewed and approved by the Science Faculty Ethics Committee (SFEC) from the University of Portsmouth.

**------------------INSERT SMTABLE 1 ABOUT HERE------------------**

## Methods

### Detailed Procedure

Participants were told that the purpose of the study was to examine how people work together as part of a team. Each dyad began the study in the same room (strangers were introduced to each other) but was then separated. Participants were randomly attributed a role (Player 1 or Player 2). In **stage 1**, participants were first asked to complete demographics questions followed by i) two personality questionnaires : the “How am I in General” [[2](#_ENREF_2)], and the Guilt and Shame Proneness Scale; [[GASP; 3](#_ENREF_3)], ii) a mood-check questionnaire [[Positive and Negative Affect Schedule scales - PANAS; 4](#_ENREF_4)], and finally iii) a friendship questionnaire [[the Unidimensional Relationship Closeness Scale, ranging from 1 low degree of closeness to 7 highly close – URCS; 5; see Sup Mat PersoQuest for details](#_ENREF_5)]. Question order was randomised between participants; questionnaires order was fix for all participants. The cooperative task (see Figure 2) was then explained and a video taken of Player 1 (control/neutral video) to provide stimuli for Player 2 (**Stage 4**). Participants were then reunited for **Stage 2**: a cooperative game where the participants had to work together to move a device from one end of the table to the other without dropping a marble [[inspired from 6](#_ENREF_6)]. The device was supporting a marble (see Figure 2) and the point of the task was to move the device back and forth, as many times as possible within 3 minutes without making the marble fall. Participants were told that at the beginning of the game, they were attributed to a shared reward of £20. However, they were informed that the shared reward would reduce each time the marble fell. Participants were also told that individual cooperative abilities and fine motor function were assessed during the task in order to determine who was responsible for the majority of marble drops. On completion of the task the participants were divided for **Stage 3**, where they completed more questionnaires [[mood-check, a friendship closeness scale, and the Dirty Dozen [DD; 7]](#_ENREF_7)]. In **Stage 4** both participants were then randomly allocated into different conditions (see below) and completed one final mood check before being asked to split the reward between their partner and themselves (see below for details; see Fig 1 for the detailed procedure).

At the end of the study, all participants were debriefed and informed of the real goal of the study. They were also reassured that their performances during the game were not assessed and that the feedback provided was fake.

**Stage 4 – Player 1.** After the game, Player 1 was informed that his/her individual performance on the task meant that they have failed the task as a team. The experimenter explained that we were monitoring concentration and motor abilities through observational analysis and that his/her concentration and motor coordination was lower than the partner’s. As a result, they had not met the threshold for the shared reward, and instead both would receive a lower payment (£15 to share instead of £20). During this feedback session, the face and upper body of Player 1 were video recorded to provide stimuli for player 2 (see below). The player was asked to split the reward (£15) between themselves and their partner however they wish to [[i.e. a dictator game, 8](#_ENREF_8)]. This money split was designed as a measure of relationship repair, potentially to mend the previous transgression [[8](#_ENREF_8)]. Half of the time, Player 1 was given an additional piece of false information. They were told that Player 2 understands that it is their fault that they failed the task and thinks it would be unfair to split the money equally (other-induced guilt). The other participants were not given any additional information here, to test the effect of spontaneously induced guilt (self-induced guilt).

**Stage 4 – Player 2.** Player 2 was allocated to one of three conditions. In condition 1, they were told that the £20 reward was reduced to £15 due to the poor performance of Player 1, but that Player 1 had already split the £15 reward in Player 1’s favour (10 for P1/5 for P2). This unfair original split was designed as a social transgression and an injustice. We wanted to measure Player 2’s propensity to rectify a social injustice by assessing the change made to this original split. They were then presented with a ‘guilty’ video of Player 1 (when Player 1 received the poor feedback from the game) and were offered the chance to change the reward split [[dictator game, 8](#_ENREF_8)]. Condition 2 was identical but the video of Player 1 was ‘neutral’ (video taken when the game was explained in Stage 1). An additional control condition (Condition 3) included the ‘guilty’ video but in the absence of any contextual information (not told that it was Player 1’s fault and not told that Player 1 had pre-split the reward; both participants shared the failure). In all conditions, Player 2 was asked to judge the emotional state of Player 1 (including how guilty they thought Player 1 looked in the video) using the PANAS [[4](#_ENREF_4)].

### Measures

**Facial expression.** Coding was conducted using the Interact© software [[9](#_ENREF_9)]. To ensure unbiased results, reliability analysis was conducted with another certified FACS coder on 22 videos out of the 215 video clips (~10%) extracted from the study videos. The agreement calculation (Wexler’s agreement) was calculated based on [Ekman, Friesen, and Hager](https://docs.google.com/document/d/1YM6nMvIrW1_7bqGf1BPa-ADPIu5rBCiaqu2j66MQoa4/edit#heading=h.46r0co2)’s Investigator’s Guide [[10](#_ENREF_10)]. Agreement between coders was 72%, considered good agreement in FACS methodology [[10](#_ENREF_10)] and considering the coding scheme used in the present study.

To reduce the FACS dataset to try and identify common and meaningful facial movements, we used a binomial exact test as criteria for exclusion - if any AU/AD was produced by fewer players than the calculated criteria, this AU/AD was removed from the dataset. The binomial exact test allowed us to keep facial movements produced significantly more than chance: if at least 62 participants produced the movement, then this movements reliably occurs across most participants and is not resulting from individual differences. We also included self-directed behaviours (face touching, neck touching, hair touching, ear touching, and scratching) and AUs identified as associated with the expression of guilt in a previous study [[AUs 4+20; see 11](#_ENREF_11)]. This left us with 18AU/ADs (out of a possible 44 observed in our data; see SMTable 2).

**------------------INSERT SMTABLE 2 ABOUT HERE------------------**

### Statistical Analysis

**Guilt induction.** To test for the success of the induction of guilt during the guilt induction task, we compared the affect data collected through the PANAS questionnaires (before vs. after induction) using a within-subjects *t*-test. We tested for a change in positive and negative affect before vs. after induction, and additionally, some specific emotional changes in guilt, shame, distress, and pride, which were all measured in the PANAS questionnaire.

**Facial expressions.** The likelihood of an action unit to be active during any communication event is likely influenced by a number of non-independent factors (e.g., information transmitted and the context, inter-individual and cultural differences, intensity of stimuli, duration of expressions, non-independence in the co-occurrence of action units). Furthermore, the likelihood of occurrence of any action unit is not independent from the likelihood of using a certain number of action units at the same time. Here, following the approach used in a previous experiment [[11](#_ENREF_11)], we used a bootstrapping approach to test whether action units differ between guilt or control videos collected during this study. In all analyses, we controlled for participants’ PoO and level of friendship of the pair.

Using this procedure, we created 1000 bootstrapped null distributions for each statistical test that have the appropriate underlying data structure and address potential problems arising from inter-individual and cultural differences. Using frame-by-frame FACS analyses, we established the frequency of occurrence for each action unit for the test condition (observed frequency) and for each action unit over all 1000 bootstraps of the control condition (expected frequency if the data would arise from the same population). Randomisation for the bootstraps was conducted on the individual level: individuals from the control condition were randomly selected with replacements. The bootstraps were conducted so that the ratio of the two places of origin and the two friendship categories (weak friendship: *if one or both players reported a friendship index 4<*; strong friendship: *both players reported a friendship index ≥ 4*) in the null condition were identical with the ratio in the test condition, to avoid results being driven by different underlying group structures. To test whether the frequency of occurrence in the test condition was significantly higher or lower than expected in the control condition, we report the z-value of the observed frequency compared to the null distribution (i.e., how many standard deviations does it differ from the mean). We assumed that the null hypothesis (the observed value for the test data is part of the same distribution that created the control condition) was rejected if the observed value was more extreme than 99% of bootstrapped values (two-sided testing). The p-value represents the likelihood of the observed frequency of an action unit in the test condition being lower or higher than the expected frequency of each bootstrap. We set our significance level at 0.01 to account for multiple testing while avoiding false rejections [[12](#_ENREF_12)]. A p-value of 0.01 and a positive z-value indicates that in 990 out of 1000 bootstrapped selections of the control data, the action unit occurred less frequently than in the test data.

We tested three questions using this approach: first, we tested the overall difference between the control condition and the guilt condition of the experiment, to see whether there were differences in the facial expressions between experimental interventions. However, there were considerable differences between individuals in their reported feeling of guilt before and after the intervention in the guilt condition.

Thus, secondly, we investigated individuals who did not show any change in reported guilt (‘weak guilt’ condition) and individuals who showed an increase in reported guilt (‘strong guilt’ condition) separately to test whether these differences in reported guilt also showed in the facial activity. We tested the two guilt conditions against each other to see if stronger reported guilt led to increased production of some action units.

Third, to test how the videos chosen by the judges as displaying guilt differed from those videos that were not judged to display guilt, we compared a) the action units in the control videos that were judged to display guilt and no guilt, and b) the action units in the guilt videos that were judged to display guilt and no guilt.

**Reward split**. We ran General Linear Models to explore which other factors could influence the rewards split made by either Player 1 or Player 2. Analyses were conducted in R v.3.6.1 [[13](#_ENREF_13)]. The amount given to the other participant was set as the response variable. As predictor variables, we set the guilt felt (Player 1) or judged (Player2), the friendship index, and the condition the participant was in (blamed vs not-blamed for Player 1; guilt video vs control video for Player 2).

We also tested which other factors (other than the facial signals) could influence the judgement of guilt. The average guilt rating of judges (range 1 – 5, mean 1.77) was set as the response variable and treated as a binary variable (low guilt: rating 1-2 on the PANAS; high guilt: rating 3-5 on the PANAS). Guilt ratings were available for 108 videos. As predictor variables, we set the condition (which video was seen: control or guilt); PoO of the judge; and friendship index.

Finally, we looked at the impact of various personality traits, as well as perceived responsibility, on reported guilt (self-reported or judged) and reward split using GLMs. The GASP scales, the Big Five items, and the annoyance felt toward self and partner were set as our predictor variables in three different models, and the reported guilt (self-reported or judged) was set as our dependent variable. We fitted the GLMs using the function glm provided by the packages lme4 and lmerTest for RStudio [[14](#_ENREF_14)].

### Results.

#### Feeling guilty (Player 1)

**Self-reported guilt.**

After receiving the feedback in Stage 4, Player 1 experienced an increase in negative affect (*M* = -0.24, *CI* = [-0.32; -0.15]; t(106) = -5.51, p < 0.001), and decrease in positive affect (*M* = 0.42, *CI* = [0.31; 0.54]; t(106) = 7.40, p < 0.001; *means and SE presented characterise the difference between the values before T2 and the values after T3 the game*). More specifically, participants reported increased guilty feelings (*M* = -0.6644, *CI* = [-0.90; -0.43]; t(106) = -5.62, p < 0.001) and shame feelings (*M* = -0.53, *CI* = [-0.72; -0.34]; t(106) = -5.6, p < 0.001), and decreased pride (*M* = 0.52, *CI* = [0.32; 0.73]; t(106) = 5.12, p < 0.001). No changes were found in self-reported distress (*M* = -0.14, *CI* = [-0.29; 0.014]; t(106) = -1.80, p = 0.075). Players reported a significantly higher level of guilt than shame after the induction task (*M* = -0.19, *CI* = [-0.37; -0.0005]; t(106) = -1.99, p = 0.05), suggesting that guilt was the primary induced emotion (see SMTable 3 for details). The level of friendship did not affect how Player 1 felt after receiving the feedback (ß = 0.042; SE = 0.068; p = 0.536).

**------------------INSERT SMTABLE 3 ABOUT HERE------------------**

**Comparison of guilt and control conditions**. The results of the bootstrap test, creating expected distributions for action units based on the control condition and comparing those with the observed distribution of action units in the guilt condition, revealed that participants in the guilt condition exhibited facial muscle activation that was significantly different from the control condition. SMTable 5 presents the summary of the comparison for the entire guilt dataset. In the upper face, AU4 was more active in the guilt condition more than in the control videos. In the lower face, AU10, AU12, and AU20 were active more often than would have been predicted based on the control videos. Most striking was the difference in the likelihoods of participants to touch their face, hair, ear, scratch, or laugh in the guilt videos. Participants in the guilt videos were significantly less likely to show activation of AU24.

**------------------INSERT SMTABLE 5 ABOUT HERE------------------**

*SMTable 5: Comparison of the full dataset for the Guilt condition (Observed Frequency) with the predicted distribution based on the Non-guilt condition (Expected Frequency) after controlling for differences in the Places of Origins and Friendship of participants. P-values denote the likelihood that the observed frequency of occurrence for an AU was more extreme than the predicted frequency. AU with significantly increased occurrence in bold, AU with significantly reduced occurrence in italics.*

**Comparison of weak guilt and strong guilt samples.** In the direct comparison between the two groups (individuals who expressed changes in feeling of guilt and those who did not; SMTable 06), individuals who reported strong feelings of guilt were more likely than expected to touch their ear and laugh (corroborating the results of comparing guilt videos with the control videos). AU1, AU2, AU4, AU14, and AU24 were significantly less likely to occur in participants who reported strong guilt.

**------------------INSERT SMTABLE 6 ABOUT HERE------------------**

SMTable 6: Comparison of the dataset for participant who reported changes in feelings of guilt (Observed Frequency) with the predicted distribution based on participants who reported no changes in feelings of guilt (Expected Frequency) after controlling for differences in the Places of Origins and Friendship of participants. P-values denote the likelihood that the observed frequency of occurrence for an AU was more extreme than the predicted frequency. AU with significantly increased occurrence in bold, AU with significantly decreased occurrence in italics.

**Personality**

We investigated the relationship between the GASP scales, the Big Five items, and annoyance, and the reported guilt. All four subscales of the GASP scale correlated positively with the reported guilt: both guilt subscales – Repair action tendency (ß = 0.425; SE = 0.159; p < 0.01), and Negative Behaviour-Evaluations (ß = 0.267; SE = 0.105; p = 0.0128); and both shame subscales – Withdrawal action tendencies (ß = 0.289; SE = 0.108; p < 0.01), and Negative Self-Evaluations (ß = 0.346; SE = 0.119; p <0.01). Moreover, we found a positive relationship with both Agreeableness (ß = 0.695; SE = 0.283; p = 0.016) and Emotional stability (ß = 0.440; SE = 0.201; p = 0.031). The level of annoyance felt toward oneself (see SMTable 4) affected greatly the level of felt guilt reported: the more annoyed they were at their performance during the game, the guiltier they felt after (ß = 0.023; SE = 0.0039; p <0.0001). The annoyance felt towards the partner (ß = 0.0048; SE = 0.0071; p = 0.502) and the attribution of blame ß = -0.14; SE = 0.17; p = 425) did not affect the level of reported self-guilt.

**------------------INSERT SMTABLE 4 ABOUT HERE------------------**

**Reward split and personality.** We investigated the relationship between the GASP scales, the Big Five items, annoyance, and the reward split. Two subscales of the GASP scale correlated positively with Player 1’s decision (see Sup Mat PersoQuest for details on GASP): one of the guilt subscales – Negative Behaviour-Evaluations (ß = 0.023; SE = 0.010; p = 0.027), and one of the shame subscales – Negative Self-Evaluations (ß = 0.027; SE = 0.011; p = 0.0204). Moreover, we found a positive relationship between money split and Conscientiousness: the higher players rated on this item, the more money they gave their partner (ß = 0.050, SE = 0.020; p = 0.0164). The level of annoyance felt toward oneself also affected the final reward split: the more annoyed players reported being at themselves, the more money they gave their partner (ß = 0.0012, SE = 0.00041; p = 0.00533). Moreover, the level of annoyance felt towards the partner affected negatively the reward split: the more annoyed they were at their partner, the less money they gave them (ß = -0.0022, SE = 0.00076; p = 0.00547). The attribution of blame did not impact the reward split (ß = 0.027, SE = 0.018; p = 0.147).

#### Perceiving guilt in others (Player 2)

**Manipulation check - felt guilt**

After receiving the feedback that Player 1 was at fault during the cooperative task failure (conditions 1 and 2), Player 2 experienced a decrease in negative affect (*M* = 0.16, *CI* = [0.046; 0.27]; t(71) = 2.84, p < 0.01), and also a decrease in positive affect (*M* = 0.28, *CI* = [0.11; 0.46]; t(71) = 3.26, p < 0.01). There was also a significant decrease in guilty feelings (*M* = 0.38, *CI* = [0.15; 0.60]; t(71) = 3.33, p < 0.01), and shame feelings (*M* = 0.24, *CI* = [0.042; 0.44]; t(70) = 2.41, p = 0.018), and a significant increase in pride (*M* = -0.34, *CI* = [-0.62; -0.055]; t(70) = -2.38, p = 0.0198). No changes were found in self-reported distress (*M* = 0.20, *CI* = [-0.013; 0.41]; t(70) = 1.87, p = 0.0656). The level of reported guilt after receiving the feedback did not differ significantly from the level of reported shame (*M* = -0.056, *CI* = [-0.21; 0.098]; t(70) = -0.73, p = 0.469; *means and SE presented characterise the difference between the values before and the values after the game*).

In the control condition (condition 3), after receiving the feedback, Player 2 experienced a decrease in negative affect (*M* = 0.12, *CI* = [0.0003; 0.24]; t(35) = 2.036, p = 0.049), a decrease in positive affect (*M* = 0.14, *CI* = [0.0038; 0.28]; t(35) = 2.087, p = 0.044) and a significant decrease in shameful feelings (*M* = 0.36, *CI* = [0.068; 0.65]; t(35) = 2.50, p = 0.0172). There were no differences in the levels of guilt, distress or pride reported after receiving the feedback (see SMTable 3).

**Judged guilt and facial movements**

**Control videos.** The control videos presented to judges that were judged as presenting guilt differ significantly from other control videos by showing more activity in AU5, AU18, AU24, face touching and scratching (SMTable 07). They also showed less activity of AU1, AU2, AU7, and AU20.

**------------------INSERT SMTABLE 7 ABOUT HERE------------------**

SMTable 7: Comparison of the non-guilt condition that were judged to reveal guilt (Observed Frequency) with the predicted distribution based on videos of the non-guilt condition that were judged to not reveal guilt (Expected Frequency) after controlling for differences in the Places of Origins. P-values denote the likelihood that the observed frequency of occurrence for an AU was more extreme than the predicted frequency. AU with significantly increased occurrence in bold, AU with significantly decreased occurrence in italics.

**Guilt videos.** The guilt videos that were judged as presenting guilt differ significantly from other guilt videos by showing more activity in AU18, AU24, and ear touching (SMTable 8). They also show less activity of AU10, AU12, AU14, and face touching.

**------------------INSERT SMTABLE 8 ABOUT HERE------------------**

SMTable 8: Comparison of the guilt condition that were judged to reveal guilt (Observed Frequency) with the predicted distribution based on videos of the guilt condition that were judged to not reveal guilt (Expected Frequency) after controlling for differences in the Places of Origins. P-values denote the likelihood that the observed frequency of occurrence for an AU was more extreme than the predicted frequency. AU with significantly increased occurrence in bold, AU with significantly decreased occurrence in italics.

**Personality**

We investigated the relationship between the GASP scales, the Big Five items, and annoyance, and the self-reported guilt, looking at all conditions together. Two subscales of the GASP scale correlated positively with Player 2 felt guilt: both shame subscales – Withdrawal action tendencies (ß = 0.16; SE = 0.072; p < 0.01), and Negative Self-Evaluations (ß = 0.17; SE = 0.069; p <0.01). The level of annoyance felt toward oneself (see SMTable 4) affected greatly the level of felt guilt reported: the more annoyed they were at their performance during the game, the guiltier they felt after (ß = 0.0018; SE = 0.0048; p = 0.0372).

**Judged guilt and personality.** We investigated the relationship between the GASP scales, the Big Five items, annoyance towards self or partner, and the judged guilt, looking at all conditions together. None of the personality traits of Player 2 influenced the way they judged Player 1’s guilt (GASP: p > 0.140; Big Five: p > 0.082). However, we found that level of annoyance felt toward oneself influenced the level of judged guilt: the more annoyed they were at their own performance during the game, the guiltier they thought Player 1 looked in the video (ß = 0.013; SE = 0.0064; p = 0.046). The annoyance felt towards their partner (ß = -0.0087; SE = 0.0078; p = 0.265) and the person to blame (ß = -0.016; SE = 0.18; p = 0.928) did not influence the judged guilt (r^2^ = 0.05).

**Reward split and personality.** We investigated the relationship between the GASP scales, the Big Five items, annoyance at their partner, and the final split, looking at all conditions together. One subscale of the GASP scale correlated negatively with Player 2’s final decision: the guilt Repair action tendencies subscale (ß = -0.027; SE = 0.012; p = 0.0228). None of the other personality data collected influenced the final decision: Big Five index, p > 0.101; annoyance at partner, ß = 0.0011; SE = 0.00087; p = 0.197; annoyance at self, ß = - -0.00061; SE = 0.00067 p = 0.365; blame, ß = 0.020; SE = 0.020; p = 0.318; r^2^ = 0.03.

**Reward split - Absence of context (control condition 3).** Player 2’s response in the control condition was examined in a GLM with friendship and cultural congruency set as our predictor variables, and the reward split as our dependent variable. Player 2 tended to give more money to their partner, keeping 7 coins on average for themselves (*M* = 7.08, *SD* = 1.34) and giving 8 coins to their partner (*M* = 7.92, *SD* = 1.44; t(35) = -1.88, p = 0.0692). Player 2’s reward split was not affected by judged guilt (ß = -0.0073, SE = 0.019, p = 0.700), or friendship (ß = 0.00101, SE = 0.0082, p = 0.903).

## References

1. Henrich J., Heine S.J., Norenzayan A. 2010 The weirdest people in the world? *Behav Brain Sci* **33**(2-3), 61-83.

2. John O.P., Naumann L.P., Soto C.J. 2008 Paradigm shift to the integrative big five trait taxonomy. *Handbook of personality: Theory and research* **3**(2), 114-158.

3. Cohen T.R., Wolf S.T., Panter A.T., Insko C.A. 2011 Introducing the GASP scale: a new measure of guilt and shame proneness. *Journal of personality and social psychology* **100**(5), 947.

4. Watson D., Clark L.A., Tellegen A. 1988 Development and validation of brief measures of positive and negative affect: the PANAS scales. *Journal of personality and social psychology* **54**(6), 1063.

5. Dibble J.L., Levine T.R., Park H.S. 2012 The Unidimensional Relationship Closeness Scale (URCS): Reliability and validity evidence for a new measure of relationship closeness. *Psychol Assessment* **24**(3), 565.

6. Miossec S., Kheddar A. 2009 Human motion in cooperative tasks: Moving object case study. In *Robotics and Biomimetics, 2008 ROBIO 2008 IEEE International Conference on* (pp. 1509-1514, IEEE.

7. Jonason P.K., Webster G.D. 2010 The Dirty Dozen: A Concise Measure of the Dark Triad. *Psychol Assessment* **22**(2), 420-432.

8. Forsythe R., Horowitz J.L., Savin N.E., Sefton M. 1994 Fairness in simple bargaining experiments. *Games and Economic behavior* **6**(3), 347-369.

9. Mangold P. 1998 Interact [computer software]. *Arnstorf, Germany: Mangold International*.

10. Ekman P., Friesen W.V., Hager J.C. 2002 Facial action coding system—investigator’s guide. *Research Nexus, Salt Lake City*.

11. Julle-Danière E., Whitehouse J., Vrij A., Gustafsson E., Waller B.M. under review Are there non-verbal signals of guilt? *Plos One*.

12. Benjamini Y., Yekutieli D. 2001 The control of the false discovery rate in multiple testing under dependency. *The annals of statistics* **29**(4), 1165-1188.

13. Team R.D. 2016 R: A Language and Environment for Statistical Computing. *R Found Stat Comput*.

14. Bates D., Mächler M., Bolker B., Walker S. 2014 Fitting linear mixed-effects models using lme4. *arXiv preprint arXiv:14065823*.
