# Supplementary material for: The social function of the feeling and expression of guilt
Source: R Soc Open Sci. 2020 Dec 9;7(12):200617. doi: 10.1098/rsos.200617 (PMC7813227; doi:10.1098/rsos.200617)
Supplement: Supplementary Material - Personality questionnaires [file rsos200617supp2.docx]

## Supplementary Material - Personality questionnaires

**Big Five – “How am I in general?”**

People’s personality is commonly measured using five main traits or domains used to estimate individual differences in thinking, feeling, and: Extraversion, Agreeableness, Conscientiousness, Negative Emotionality (alternatively labelled Neuroticism vs. Emotional Stability), and OpenMindedness (alternatively labelled Openness to Experience). We chose to measure those personality traits using a questionnaire made of 44 items asking people to reflect on different aspect of their behaviours or personalities in their daily life– “How am I in general?”. On a scale from 1 (*Disagree strongly*) to 5 (*Agree strongly*), participants had to indicate how the presented characteristics applied to them.

**Scoring.**  Reverse score the items labelled “R” and compute the scale score as the mean of the following items:

- Extraversion: 1, 6R, 11, 16, 21R, 26, 31R, 36
- Agreeableness: 2R, 7, 12R, 17, 22, 27R, 32, 37R, 42
- Conscientiousness: 3, 8R, 12, 19R, 23R, 28, 33, 38, 43R
- Neuroticism: 4, 9R, 14, 19, 24R, 29, 34R, 39
- Openness: 5, 10, 15, 20, 25, 30, 35R, 40, 41R, 44

*Here are a number of characteristics that may or may not apply to you. For example, do you agree that you are someone who likes to spend time with others? Please write a number next to each statement to indicate the extent to which you agree or disagree with that statement.*

*I am someone who…*

1. *Is talkative*
2. *Tends to find fault with others*
3. *Does a thorough jog*
4. *Is depressed, blue*
5. *Is original*
6. *Is reserved,*
7. *Is helpful and unselfish with others*
8. *Can be somewhat careless*
9. *Is relaxed, handles stress well*
10. *Is curious about many different things*
11. *Is full of energy*
12. *Starts quarrels with others*
13. *Is a reliable worker*
14. *Can be tense*
15. *Is ingenious, a deep thinker*
16. *Generated a lot of enthusiasm*
17. *Has a forgiving nature*
18. *Tends to be disorganised*
19. *Worries a lot*
20. *Has an active imagination*
21. *Tends to be quiet*
22. *Is generally trusting*
23. *Tends to be lazy*
24. *Is emotionally stable, not easily upset*
25. *Is inventive*
26. *Has an assertive personality*
27. *Can be cold and aloof*
28. *Perseveres until the task is finished*
29. *Can be moody*
30. *Values artistic, aesthetic experiences*
31. *Is sometimes shy, inhibited*
32. *Is considerate and kind to almost everyone*
33. *Does things efficiently*
34. *Remains calm in tense situations*
35. *Prefers work that is routine*
36. *Is outgoing, sociable*
37. *Is sometimes rude to others*
38. *Makes plans and follows through with them*
39. *Gets nervous easily*
40. *Likes to reflect, play with ideas*
41. *Has few artistic interests*
42. *Likes to cooperate with others*
43. *Is easily distracted*
44. *Is sophisticated in art, music, or literature*

**Dirty Dozen**

The Dirty Dozen is a 12-item self-report scale measuring the Dark Triad: narcissism (e.g., the need for attention, associated with high self-perception), psychopathy (e.g., lack of remorse, callous behaviour tendencies), and Machiavellianism (e.g., manipulating people, unethical behaviours to reach one goal). The Dark Triad refer to three undesirable personality trait, often associated with social shortcoming and unethical decision-making. Using a scale ranging from 1 (*Strongly disagree*) to 5 (*Strongly agree*), participants had to indicate how much they agreed with each of the 12 statements. We used it to see how those personality traits could influence one self-reported guilt or judgement of guilt, as well as behavioural outcomes.

**Scoring.** Scores are computed by averaging the 4 items in each subscale.

- Machiavellianism: 1-4
- Narcissism: 5-8
- Psychopathy: 9-12

*1. I tend to manipulate others to get my way*

*2. I have used deceit or lied to get my way*

*3. I have use flattery to get my way*

*4. I tend to exploit others towards my own end.*

*5. I tend to lack remorse.*

*6. I tend to be unconcerned with the morality of my actions.*

*7. I tend to be callous or insensitive*

*8. I tend to be cynical.*

*9. I tend to want others to admire me.*

*10. I tend to want others to pay attention to me.*

*11. I tend to seek prestige or status*

*12. I tend to expect special favours from others.*

**PANAS**

The Positive and Negative Affect Schedule scales are composed of two 10-item mood scales. Each scale is made of 10 emotion words that are most commonly used when describing an emotional state. The positive scale is composed of a*ctive, alert, attentive, determined, enthusiastic, excited, inspired, interested,* ***proud****,* and *strong*; the negative scale is composed of *afraid,* ***ashamed****,* ***distressed****,* ***guilty****, hostile, irritable, jittery, nervous, scared*, and *upset.* The Positive Affect Score is calculated by adding the scores of all the positive items; the Negative Affect Score is calculated by adding the scores of all the negative items. When used with short-term instructions (e.g., *right now*), the PANAS scales measure fluctuation in mood and emotional states. In this study, we also looked at the evolution across time of the Positive and Negative Affect Scores as well as the changes in specific emotional state (in bold previously: proud, ashamed, distressed, and guilty). We used the PANAS to control for the efficiency of our experimental settings: does Player 1 feel generally worst and report an increase of guilty feeling after receiving he feedback of the game compare to before? Does receiving positive feedback regarding one’s performance increase the positive mood of Player 2 and reduced it level of guilt compare to right after the game?

**GASP**

The Guilt and Shame Proneness scale measures individual differences in the propensity to experience guilt and shame. The GASP contains two guilt subscales: negative behaviour-evaluations (i.e., feeling bad about how you acted, useful to detect potential unethical decision making) and repair action tendencies (i.e., behaviours and behavioural tendencies); and two shame subscales: negative self-evaluations (i.e., feeling bad about yourself, measuring the moral, prosocial aspect of shame proneness) and withdrawal action tendencies (i.e., action tendencies and behaviours).

Participants are presented with 16 items and asked, using a scale ranging from 1 (*Very Unlikely*) to 7 (*Very Likely*), how well each situation applied to them. We used the GASP in order to assess the relationship between guilt proneness and self-reported guilt as well as judged guilt. We wanted to investigate how one’s propensity to experience guilt translated in the present setting.

**GASP scoring.** The GASP is scored by averaging the four items in each subscale.

- Guilt-Negative-Behavior-Evaluation (NBE): 1, 9, 14, 16
- Guilt-Repair: 2, 5, 11, 15
- Shame-Negative-Self-Evaluation (NSE): 3, 6, 10, 13
- Shame-Withdraw: 4, 7, 8, 12

*In this questionnaire you will read about situations that people are likely to encounter in day-to-day life, followed by common reactions to those situations. As you read each scenario, try to imagine yourself in that situation. Then indicate the likelihood that you would react in the way described.*

*_______ 1. After realizing you have received too much change at a store, you decide to keep it because the salesclerk doesn't notice. What is the likelihood that you would feel uncomfortable about keeping the money?*

*_______ 2. You are privately informed that you are the only one in your group that did not make the honor society because you skipped too many days of school. What is the likelihood that this would lead you to become more responsible about attending school?*

*_______ 3. You rip an article out of a journal in the library and take it with you. Your teacher discovers what you did and tells the librarian and your entire class. What is the likelihood that this would make you would feel like a bad person?*

*_______ 4. After making a big mistake on an important project at work in which people were depending on you, your boss criticizes you in front of your coworkers. What is the likelihood that you would feign sickness and leave work?*

*_______ 5. You reveal a friend’s secret, though your friend never finds out. What is the likelihood that your failure to keep the secret would lead you to exert extra effort to keep secrets in the future?*

*_______ 6. You give a bad presentation at work. Afterwards your boss tells your coworkers it was your fault that your company lost the contract. What is the likelihood that you would feel incompetent?*

*_______ 7. A friend tells you that you boast a great deal. What is the likelihood that you would stop spending time with that friend?*

*_______ 8. Your home is very messy and unexpected guests knock on your door and invite themselves in. What is the likelihood that you would avoid the guests until they leave?*

*_______ 9. You secretly commit a felony. What is the likelihood that you would feel remorse about breaking the law?*

*10. You successfully exaggerate your damages in a lawsuit. Months later, your lies are discovered and you are charged with perjury. What is the likelihood that you would think you are a despicable human being?*

*_______ 11. You strongly defend a point of view in a discussion, and though nobody was aware of it, you realize that you were wrong. What is the likelihood that this would make you think more carefully before you speak?*

*_______ 12. You take office supplies home for personal use and are caught by your boss. What is the likelihood that this would lead you to quit your job?*

*_______ 13. You make a mistake at work and find out a coworker is blamed for the error. Later, your coworker confronts you about your mistake. What is the likelihood that you would feel like a coward?*

*_______ 14. At a coworker’s housewarming party, you spill red wine on their new creamcolored carpet. You cover the stain with a chair so that nobody notices your mess. What is the likelihood that you would feel that the way you acted was pathetic?*

*_______ 15. While discussing a heated subject with friends, you suddenly realize you are shouting though nobody seems to notice. What is the likelihood that you would try to act more considerately toward your friends?*

*_______ 16. You lie to people but they never find out about it. What is the likelihood that you would feel terrible about the lies you told?*

**URCS**

The Unidimensional Relationship Closeness Scale is a 12-item self-report scale measuring closeness of social and personal relationships. It is used to measure the strength of the emotional bond between two individuals, and applied to both romantic and friendly relationships. Using a scale ranging from 1 (*Strongly Disagree*) to 7 (*Strongly Agree*), participants had to indicate how each statement applied to them. The URCS conceptualise closeness as a continuum, which allowed us to include this measure as a variable in our models. We used this score to investigate how closeness affected one’s behavioural response, both as a guilty part or as a victim of one’s wrongdoing.

**Scoring.** The items are averaged to create a single overall closeness score.

*The following questions refer to your relationship with* ***the other player****. Please think about your relationship with* ***the other player*** *when responding to the following questions.*

*1. My relationship with* ***the other player*** *is close.*

*2. When we are apart, I miss* ***the other player*** *a great deal.*

*3.* ***The other player*** *and I disclose important personal things to each other.*

*4. T****he other player*** *and I have a strong connection.*

*5.* ***The other player*** *and I want to spend time together.*

*6. I’m sure of my relationship with* ***the other player****.*

*7.* ***The other player*** *is a priority in my life.*

*8.* ***The other player*** *and I do a lot of things together.*

*9. When I have free time, I choose to spend it alone with* ***the other player****.*

*10. I think about* ***the other player*** *a lot.*

*11. My relationship with* ***the other player*** *is important in my life.*

*12. I consider* ***the other player*** *when making important decision*
